# 3-Allyl-1-methyl-1*H*-benzotriazol-3-ium iodide

**DOI:** 10.1107/S1600536812035611

**Published:** 2012-08-23

**Authors:** Nabeel H. Buttrus, Assim A. Sabah, Amer A. Taqa, Ulli Englert

**Affiliations:** a Chemistry Department, College of Science, Mosul University, Iraq; b Science Department, College of Basic Education, Mosul University, Iraq; c DEPS Department, College of Dentistry, Mosul University, Iraq; d Institut für Anorganische Chemie, RWTH Aachen University, Landoltweg 1, 52074 Aachen, Germany

## Abstract

In the crystal structure of 1-methyl-3-allyl benzotriazolium iodide, C_10_H_12_N_3_^+^·I^−^, centrosymmetric dimers of coplanar cations are π-stacked with an inter­planar distance of 3.453 (6) Å. The iodide anions are situated above and below the formally positive charged triazolium rings.

## Related literature

For information on the Cambridge Structural Database, see: Allen (2002 ▶). For structural investigations of related compounds, see: Boche *et al.* (1996 ▶); Mouhib *et al.* (2011 ▶). For general information on π-stacking, see: Wright (1995 ▶).
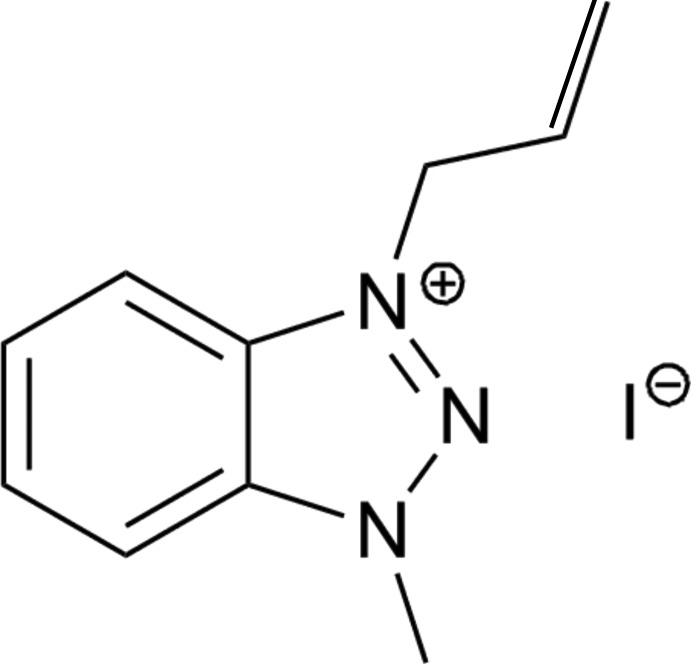


## Experimental

### 

#### Crystal data

C_10_H_12_N_3_^+^·I^−^
*M* *_r_* = 301.13
Triclinic, 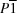

*a* = 7.8839 (12) Å
*b* = 8.2265 (14) Å
*c* = 9.9957 (17) Å
α = 114.093 (2)°
β = 104.033 (15)°
γ = 92.201 (13)°
*V* = 567.20 (16) Å^3^
*Z* = 2
Mo *K*α radiation
μ = 2.79 mm^−1^
*T* = 100 K
0.39 × 0.04 × 0.01 mm

#### Data collection

Bruker SMART CCD area-detector diffractometer
Absorption correction: multi-scan (*SADABS*; Sheldrick, 1996 ▶) *T*_min_ = 0.409, *T*_max_ = 0.972
7816 measured reflections
2798 independent reflections
2503 reflections with *I* > 2σ(*I*)
*R*_int_ = 0.089

#### Refinement

*R*[*F* ^2^ > 2σ(*F* ^2^)] = 0.035
*wR*(*F* ^2^) = 0.066
*S* = 0.96
2798 reflections
128 parameters
H-atom parameters constrained
Δρ_max_ = 1.58 e Å^−3^
Δρ_min_ = −1.35 e Å^−3^

### 

Data collection: *SMART* (Bruker, 2001 ▶); cell refinement: *SAINT-Plus* (Bruker, 1999 ▶); data reduction: *SAINT-Plus* (Bruker, 1999 ▶); program(s) used to solve structure: *SHELXS97* (Sheldrick, 2008 ▶); program(s) used to refine structure: *SHELXL97* (Sheldrick, 2008 ▶); molecular graphics: *PLATON* (Spek, 2009 ▶); software used to prepare material for publication: *SHELXL97*.

## Supplementary Material

Crystal structure: contains datablock(s) global, I. DOI: 10.1107/S1600536812035611/im2367sup1.cif

Structure factors: contains datablock(s) I. DOI: 10.1107/S1600536812035611/im2367Isup2.hkl

Supplementary material file. DOI: 10.1107/S1600536812035611/im2367Isup3.cml

Additional supplementary materials:  crystallographic information; 3D view; checkCIF report

## Figures and Tables

**Fig. 1.**
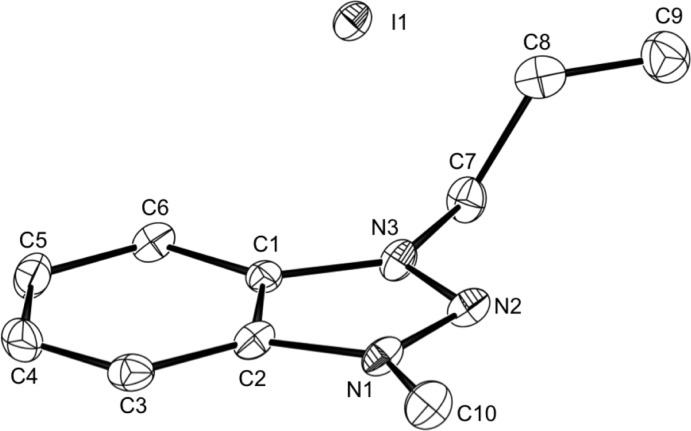
: Displacement ellipsoid plot (Spek, 2009) of the asymmetric unit of 1 with thermal ellipsoids at the 50% probability level; H atoms have been omitted.

**Fig. 2.**
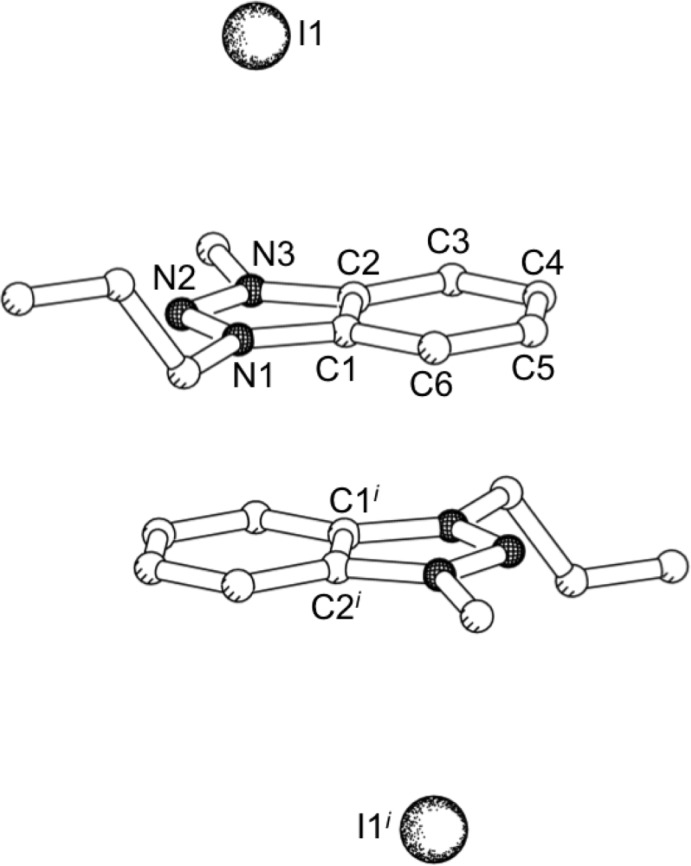
: Packing of the title compound: Two π-stacked cations related by inversion (i = 1 - x,-y,1 - z) and two counter anions are shown; H atoms have been omitted for clarity.

## supplementary crystallographic information

### Comment

The asymmetric unit of the title compound 1 (Fig. 1) comprises an organic cation and an iodide anion in general positions. The heteroaromatic cation is planar within error, with a maximum deviation of 0.008 (3) Å from the least-squares plane for the nitrogen atoms N1 and N2. Neighbouring cations are related by inversion and hence coplanar for reasons of symmetry. The shortest intermolecular interaction amounts to C2···C2^i^ = 3.453 (6) Å (i = 1 - *x*,-*y*,1 - *z*). Due to the antiparallel arrangement of the coplanar benzotriazolium cations, the formally positive part of each heteroaromatic system is located on top of the carbocyclic moiety of its neighbour, thus ensuring both efficient space filling and efficient dipole matching for the π-stacking (Wright, 1995). An iodide counter anion is located 3.5172 (3) Å above and below each triazolium ring of such a cation pair. This packing motif is shown in Fig. 2. Shortest interactions between the hydrogen atoms of neighbouring stacks and iodide anions amount to 3.02 Å for I1···H7B^ii^ (ii = *x*,1 + *y*,*z*). No relevant interhalide contacts occur, all being longer than 5 Å. Eight other benzotriazolium salts have been documented (Version 1.13, including the updates of August 2011) in the CSD data base (Allen, 2002), among them the closely related dimethylbenzotriazolium iodide studied by Boche *et al.* (1996). According to the database, the average N–N distance in the heteroaromatic five membered ring is 1.316 Å (min 1.300, max 1.338 Å); we find values of 1.309 (4) Å for N1—N2 and 1.322 (4) Å for N2—N3. The interatomic distance of 1.321 (5) Å of the allylic double bond C8—C9 closely matches the result recently obtained for the corresponding bond in allyl acetate where an interatomic distance of 1.3257 (18) Å was found by high-resolution X-ray diffraction (Mouhib *et al.*, 2010).

### Experimental

To a solution of benzotriazole (1.19 g, 0.01 mol) in 10 ml of EtOH, first CH_3_I (0.62 ml, 0.01 mol) and then 10 ml of 10% aqueous KOH were added. The mixture was refluxed for 1h. Then allyl chloride (5 ml) was added and refluxing was continued for 1h. The reaction mixture was extracted with n-hexane (3-5) times, in order to remove the excess of CH_3_I . The mixture was filtered and the solvent removed under vacuum. The residue was crystallized from ethanol to give yellow crystals (yield 75%). M.p. 148-150 °C. Elemental analysis: found C: 39.56; H: 4.40; N: 13.70, Calcd. C: 39.89; H: 4.02; N: 13.95 %.

### Refinement

H atoms were treated as riding with C_aryl_—H and C_olefin_—H 0.95 Å, *U*_iso_(H) = 1.2*U*_eq_(C); C_methylene_—H 0.99 Å, *U*_iso_(H) = 1.2*U*_eq_(C) and C_methyl_—H 0.98 Å, *U*_iso_(H) = 1.5*U*_eq_(C).

### Crystal data

**Table:**

C_10_H_12_N_3_^+^·I^−^	*Z* = 2
*M**_r_* = 301.13	*F*(000) = 292
Triclinic, *P*1	*D*_x_ = 1.763 Mg m^−^^3^
Hall symbol: -P 1	Mo *K*α radiation, λ = 0.71073 Å
*a* = 7.8839 (12) Å	Cell parameters from 2049 reflections
*b* = 8.2265 (14) Å	θ = 2.3–25.1°
*c* = 9.9957 (17) Å	µ = 2.79 mm^−^^1^
α = 114.093 (2)°	*T* = 100 K
β = 104.033 (15)°	Rod, yellow
γ = 92.201 (13)°	0.39 × 0.04 × 0.01 mm
*V* = 567.20 (16) Å^3^	

### Data collection

**Table:**

Bruker SMART CCD area-detector diffractometer	2798 independent reflections
Radiation source: Incoatec microsource	2503 reflections with *I* > 2σ(*I*)
Multilayer optics monochromator	*R*_int_ = 0.089
ω scans	θ_max_ = 28.3°, θ_min_ = 2.3°
Absorption correction: multi-scan (*SADABS*; Sheldrick, 1996)	*h* = −10→10
*T*_min_ = 0.409, *T*_max_ = 0.972	*k* = −10→10
7816 measured reflections	*l* = −13→13

### Refinement

**Table:**

Refinement on *F*^2^	Primary atom site location: structure-invariant direct methods
Least-squares matrix: full	Secondary atom site location: difference Fourier map
*R*[*F*^2^ > 2σ(*F*^2^)] = 0.035	Hydrogen site location: inferred from neighbouring sites
*wR*(*F*^2^) = 0.066	H-atom parameters constrained
*S* = 0.96	*w* = 1/[σ^2^(*F*_o_^2^) + (0.006*P*)^2^] where *P* = (*F*_o_^2^ + 2*F*_c_^2^)/3
2798 reflections	(Δ/σ)_max_ = 0.002
128 parameters	Δρ_max_ = 1.58 e Å^−^^3^
0 restraints	Δρ_min_ = −1.35 e Å^−^^3^

### Special details

**Table:**

Geometry. All e.s.d.'s (except the e.s.d. in the dihedral angle between two l.s. planes) are estimated using the full covariance matrix. The cell e.s.d.'s are taken into account individually in the estimation of e.s.d.'s in distances, angles and torsion angles; correlations between e.s.d.'s in cell parameters are only used when they are defined by crystal symmetry. An approximate (isotropic) treatment of cell e.s.d.'s is used for estimating e.s.d.'s involving l.s. planes.
Refinement. Refinement of *F*^2^ against ALL reflections. The weighted *R*-factor *wR* and goodness of fit *S* are based on *F*^2^, conventional *R*-factors *R* are based on *F*, with *F* set to zero for negative *F*^2^. The threshold expression of *F*^2^ > σ(*F*^2^) is used only for calculating *R*-factors(gt) *etc*. and is not relevant to the choice of reflections for refinement. *R*-factors based on *F*^2^ are statistically about twice as large as those based on *F*, and *R*- factors based on ALL data will be even larger.

### Fractional atomic coordinates and isotropic or equivalent isotropic displacement parameters (Å^2^)

**Table:**

	*x*	*y*	*z*	*U*_iso_*/*U*_eq_	
N1	0.7209 (4)	0.1330 (4)	0.4718 (3)	0.0164 (7)	
N2	0.7597 (4)	−0.0107 (4)	0.3708 (3)	0.0176 (7)	
N3	0.6104 (4)	−0.0914 (4)	0.2612 (3)	0.0147 (7)	
C1	0.4725 (5)	0.0022 (5)	0.2899 (4)	0.0140 (8)	
C2	0.5465 (5)	0.1512 (5)	0.4296 (4)	0.0148 (8)	
C3	0.4457 (5)	0.2804 (5)	0.4962 (4)	0.0187 (8)	
H3	0.4955	0.3832	0.5906	0.022*	
C4	0.2706 (5)	0.2495 (5)	0.4171 (4)	0.0211 (9)	
H4	0.1965	0.3331	0.4589	0.025*	
C5	0.1956 (5)	0.0978 (5)	0.2754 (4)	0.0199 (9)	
H5	0.0733	0.0831	0.2252	0.024*	
C6	0.2947 (5)	−0.0288 (5)	0.2084 (4)	0.0168 (8)	
H6	0.2454	−0.1304	0.1131	0.020*	
C7	0.6077 (5)	−0.2619 (5)	0.1295 (4)	0.0177 (8)	
H7A	0.4846	−0.3094	0.0634	0.021*	
H7B	0.6502	−0.3519	0.1665	0.021*	
C8	0.7214 (5)	−0.2358 (5)	0.0381 (4)	0.0205 (9)	
H8	0.7091	−0.1396	0.0089	0.025*	
C9	0.8387 (5)	−0.3421 (6)	−0.0034 (5)	0.0271 (10)	
H9A	0.8527	−0.4390	0.0250	0.033*	
H9B	0.9091	−0.3218	−0.0615	0.033*	
C10	0.8626 (5)	0.2617 (5)	0.6012 (4)	0.0220 (9)	
H10A	0.9640	0.2009	0.6201	0.033*	
H10B	0.8201	0.3103	0.6922	0.033*	
H10C	0.8987	0.3604	0.5781	0.033*	
I1	0.77374 (3)	0.33493 (3)	0.22631 (3)	0.01529 (8)	

### Atomic displacement parameters (Å^2^)

**Table:**

	*U*^11^	*U*^22^	*U*^33^	*U*^12^	*U*^13^	*U*^23^
N1	0.0169 (18)	0.0148 (17)	0.0179 (17)	0.0011 (13)	0.0009 (14)	0.0098 (14)
N2	0.0197 (19)	0.0169 (18)	0.0164 (17)	0.0031 (14)	0.0022 (14)	0.0092 (15)
N3	0.0154 (17)	0.0100 (16)	0.0167 (16)	0.0018 (12)	0.0015 (13)	0.0056 (14)
C1	0.019 (2)	0.0129 (19)	0.0162 (19)	0.0060 (15)	0.0081 (16)	0.0097 (16)
C2	0.014 (2)	0.0121 (19)	0.0166 (19)	−0.0022 (15)	−0.0004 (15)	0.0079 (16)
C3	0.027 (2)	0.015 (2)	0.0168 (19)	0.0045 (17)	0.0080 (17)	0.0081 (17)
C4	0.027 (2)	0.018 (2)	0.024 (2)	0.0103 (17)	0.0139 (18)	0.0103 (18)
C5	0.017 (2)	0.022 (2)	0.025 (2)	0.0064 (16)	0.0047 (17)	0.0136 (19)
C6	0.019 (2)	0.015 (2)	0.0153 (19)	0.0016 (16)	0.0012 (16)	0.0071 (16)
C7	0.019 (2)	0.013 (2)	0.019 (2)	0.0038 (15)	0.0043 (16)	0.0047 (17)
C8	0.026 (2)	0.014 (2)	0.017 (2)	−0.0011 (16)	0.0033 (17)	0.0052 (17)
C9	0.023 (2)	0.031 (3)	0.025 (2)	0.0036 (19)	0.0062 (18)	0.010 (2)
C10	0.018 (2)	0.022 (2)	0.019 (2)	−0.0017 (17)	−0.0013 (17)	0.0061 (18)
I1	0.01635 (15)	0.01309 (14)	0.01588 (14)	0.00263 (10)	0.00268 (10)	0.00672 (11)

### Geometric parameters (Å, º)

**Table:**

N1—N2	1.309 (4)	C5—C6	1.375 (5)
N1—C2	1.370 (5)	C5—H5	0.9500
N1—C10	1.460 (4)	C6—H6	0.9500
N2—N3	1.322 (4)	C7—C8	1.492 (5)
N3—C1	1.376 (4)	C7—H7A	0.9900
N3—C7	1.476 (4)	C7—H7B	0.9900
C1—C2	1.394 (5)	C8—C9	1.321 (5)
C1—C6	1.394 (5)	C8—H8	0.9500
C2—C3	1.396 (5)	C9—H9A	0.9500
C3—C4	1.370 (5)	C9—H9B	0.9500
C3—H3	0.9500	C10—H10A	0.9800
C4—C5	1.417 (5)	C10—H10B	0.9800
C4—H4	0.9500	C10—H10C	0.9800
			
N2—N1—C2	112.4 (3)	C5—C6—C1	115.4 (3)
N2—N1—C10	119.4 (3)	C5—C6—H6	122.3
C2—N1—C10	127.7 (3)	C1—C6—H6	122.3
N1—N2—N3	105.9 (3)	N3—C7—C8	111.4 (3)
N2—N3—C1	111.9 (3)	N3—C7—H7A	109.3
N2—N3—C7	119.8 (3)	C8—C7—H7A	109.3
C1—N3—C7	128.3 (3)	N3—C7—H7B	109.3
N3—C1—C2	104.8 (3)	C8—C7—H7B	109.3
N3—C1—C6	132.4 (3)	H7A—C7—H7B	108.0
C2—C1—C6	122.8 (3)	C9—C8—C7	122.1 (4)
N1—C2—C1	105.0 (3)	C9—C8—H8	118.9
N1—C2—C3	133.4 (4)	C7—C8—H8	118.9
C1—C2—C3	121.6 (4)	C8—C9—H9A	120.0
C4—C3—C2	115.8 (4)	C8—C9—H9B	120.0
C4—C3—H3	122.1	H9A—C9—H9B	120.0
C2—C3—H3	122.1	N1—C10—H10A	109.5
C3—C4—C5	122.5 (4)	N1—C10—H10B	109.5
C3—C4—H4	118.7	H10A—C10—H10B	109.5
C5—C4—H4	118.7	N1—C10—H10C	109.5
C6—C5—C4	121.9 (4)	H10A—C10—H10C	109.5
C6—C5—H5	119.1	H10B—C10—H10C	109.5
C4—C5—H5	119.1		
